# Abundance of Early Embryonic Primordial Germ Cells Promotes Zebrafish Female Differentiation as Revealed by Lifetime Labeling of Germline

**DOI:** 10.1007/s10126-019-09874-1

**Published:** 2019-01-22

**Authors:** Ding Ye, Lin Zhu, Qifeng Zhang, Feng Xiong, Houpeng Wang, Xiaosi Wang, Mudan He, Zuoyan Zhu, Yonghua Sun

**Affiliations:** 1 State Key Laboratory of Freshwater Ecology and Biotechnology, Institute of Hydrobiology, The Innovative Academy of Seed Design, Chinese Academy of Sciences, Wuhan, 430072 China; 2 College of Advanced Agricultural Sciences, University of Chinese Academy of Sciences, Beijing, 100049 China

**Keywords:** *piwil1*, Spermatogenesis, Oogenesis, Primordial germ cell, Spermatogonia, Oogonia

## Abstract

Teleost sex differentiation largely depends on the number of undifferentiated germ cells. Here, we describe the generation and characterization of a novel transgenic zebrafish line, *Tg*(*piwil1*:*egfp-UTRnanos3*)^*ihb327Tg*^, which specifically labels the whole lifetime of germ cells, i.e., from primordial germ cells (PGCs) at shield stage to the oogonia and early stage of oocytes in the ovary and to the early stage of spermatogonia, spermatocyte, and spermatid in the testis. By using this transgenic line, we carefully observed the numbers of PGCs from early embryonic stage to juvenile stage and the differentiation process of ovary and testis. The numbers of PGCs became variable at as early as 1 day post-fertilization (dpf). Interestingly, the embryos with a high amount of PGCs mainly developed into females and the ones with a low amount of PGCs mainly developed into males. By using transient overexpression and transgenic induction of PGC-specific *bucky ball* (*buc*), we further proved that induction of abundant PGCs at embryonic stage promoted later ovary differentiation and female development. Taken together, we generate an ideal transgenic line *Tg*(*piwil1*:*egfp-UTRnanos3*)^*ihb327Tg*^ which can visualize zebrafish germline for a lifetime, and we have utilized this line to study germ cell development and gonad differentiation of teleost and to demonstrate that the increase of PGC number at embryonic stage promotes female differentiation.

## Introduction

In a large amount of teleosts, the number of undifferentiated germ cells strongly contribute to sex differentiation and sexual dimorphism. For instance, in zebrafish (*Danio rerio*), medaka (*Oryzias latipes*), Nile tilapia (*Oreochromis niloticus*), three-spined stickleback (*Gasterosteus aculeatus*), and even the gynogenetic all-female fish, *Carassius gibelio*, depletion of primordial germ cells usually leads to all-male development (Houwing et al. 2007; Lewis et al. 2008; Li et al. 2017; Saito et al. 2007; Siegfried and Nusslein-Volhard 2008). Previous studies have shown that zebrafish, although possesses a polygenetic sex determination system, shows different germ cell proliferations between definitive male and definitive female in larval stage at 14 days post-fertilization (dpf) (Liew et al. 2012; Tong et al. 2010; Tzung et al. 2015). A strong male-biased sex ratio was observed in primordial germ cell (PGC)-less population, indicating that a threshold number of PGCs is required for ovarian differentiation (Tzung et al. 2015). Transplantation of a single PGC into a germ cell-deficient embryos generates males exclusively in zebrafish (Saito et al. 2008). However, whether the initial PGC number at early embryonic stage contributes to zebrafish sex differentiation remains largely unknown.

The gonadal differentiation process of teleost has been thoroughly described using zebrafish as a model system. Zebrafish gonad in 17–21 dpf larva is generally considered to be undifferentiated (Rodriguez-Mari et al. 2005; Tzung et al. 2015; Wang et al. 2007). Primary testis and ovary are morphologically and molecularly distinguishable at around 30 dpf (Rodriguez-Mari et al. 2005). During testis differentiation, a “juvenile ovary-to-testis” transformation process usually occurs from 30 to 40 dpf (Maack and Segner 2003; Wang et al. 2007), which is accompanied by the Tp53-mediated apoptosis of oocytes (Rodriguez-Mari et al. 2010; Uchida et al. 2002). The cysts containing spermatogonia, spermatocytes, and spermatids can be observed in testes of males at 2 months post-fertilization (mpf) (Leu and Draper 2010; Maack and Segner 2003). The differentiation of ovary is considered to start earlier and to last for longer than that of the testis. The perinuclear oocytes (stage 1B) can be visible from 25 to 40 dpf (Lau et al. 2016; Siegfried and Nusslein-Volhard 2008), the cortical alveolar oocytes (stage II) are identical from 50 dpf (Krovel and Olsen 2004), and the matured oocytes form at 3 mpf (Yang et al. 2017). However, most of the previous results rely on chemical staining or immunostaining on gonad sections; the gonad development of zebrafish has never been investigated at the intact-gonad level with high resolution for a lifetime duration, which largely relies on a transgenic line labeling the whole lifetime of germ cells.

Several transgenic lines have been generated to visualize different types of germ cells at different stages of zebrafish. For undifferentiated germ cells, the *askops* (*kop*) promoter has been used to label the migrating PGCs until 3 dpf (Blaser et al. 2005), the *Tg*(*ddx4*:*ddx4-EGFP*)^*zf45Tg*^ (previous name: *Tg*(*vasa:EGFP*)) can be used to label PGCs from 1 dpf (Krovel and Olsen 2002), the *piwil1* promoter in *Tg*(*piwil1*:*EGFP*)^*uc1Tg*^ (previous name: *Tg*(*ziwi*:*EGFP*)) can specifically drive EGFP expression in the undifferentiated germ cells after 7 dpf (Leu and Draper 2010), and by using a Gal4/UAS system and the *kop* promoter, our previous study has shown that the undifferentiated germ cells could be visible from the shield stage to 25 dpf (Xiong et al. 2013). For female germ cells, the *Tg*(*-0.5zp3b*:*GFP*)^*m1032Tg*^ (previous name: *Tg*(*zpc0.5*:*EGFP*)) labels all stages of oocytes (Onichtchouk et al. 2003), the *vasa* promotor can be used to label the early stages of oocyte but not oogonia (pre-meiotic germ cells) (Krovel and Olsen 2004; Leu and Draper 2010; Wang et al. 2007), and *Tg*(*piwil1*:*EGFP*)^*uc1Tg*^ could specifically label the oogonia and oocytes (Leu and Draper 2010), whereas the *Tg*(*alk8*:*EGFP*) only labels the early chromatin nucleolus-stage oocytes (stage IA oocytes) (Payne-Ferreira et al. 2004). For male germ cells, both *Tg*(*ddx4*:*ddx4-EGFP*)^*zf45Tg*^ and *Tg*(*piwil1*:*EGFP*)^*uc1Tg*^ label the spermatogonia and spermatocytes (Leu and Draper 2010). To the best of our knowledge, there is no report of transgenic fish labeling the whole lifetime of germ cells, from the early PGCs to the fully differentiated gametes.

In order to accomplish a lifetime visualization of zebrafish germ cells, early post-transcription regulation elements like *nanos3 *3′-UTR and later transcriptional regulation sequences such as *piwil1* promoter might be coordinately combined. Piwil1 belongs to Argonaute protein family which interacts with Piwi-interacting RNA (piRNA) to mediate the post-transcriptional regulation of mRNA and transposon silencing in the animal germ line (Houwing et al. 2007), and a 4.7-kb promoter region could direct the expression of EGFP in germ cells from 7 dpf (Leu and Draper 2010). Nanos3 is one of the germ plasm components and is essential for maintaining oocyte production (Draper et al. 2007; Kosaka et al. 2007). During early embryogenesis, the specific region of 3′-UTR of *nanos3* can be recognized by the miR430 which mediate maternal mRNA degradation (Mickoleit et al. 2011; Mishima et al. 2006), and Dnd1 can bind to the Uracil-rich region of 3′-UTR of *nanos3* to protect the mRNA from miR430-mediated degradation in PGCs (Kedde et al. 2007; Mishima et al. 2006). Thus, the *nanos3* 3′-UTR has been widely used to mediated PGC-specific gene expression in either transient overexpression experiment or transgenic study (Saito et al. 2006; Xiong et al. 2013).

In this study, by using a combination of the *piwil1* promoter and *nanos3* 3′-UTR, we successfully generated the transgenic zebrafish line, *Tg*(*piwil1*:*egfp-UTRnanos3*)^*ihb327Tg*^, which could label the whole lifetime of zebrafish germ cells, from early PGCs to oocytes in females and spermatids in males. Furthermore, we carefully traced the development of gonad in fish with different numbers of PGCs from the embryonic stage until adulthood, and proved that the numbers of PGCs became variable at as early as 1 dpf and abundant PGCs at embryonic stage promoted later ovary differentiation and female development.

## Materials and Methods

### Zebrafish

Embryos were obtained from the natural mating of zebrafish of the AB genetic background (from the China Zebrafish Resource Center, CZRC, Wuhan, China; Web: http://zfish.cn) and maintained, raised, and staged as previously described (Kimmel et al. 1995). The density of fish is 50 larvae/L for age from 5 dpf to 15 dpf, 20 fish/L for age 15 dpf to 1 mpf, 10 fish/L for age from 1 mpf to 2 mpf, and 5 fish/L for age older than 2 mpf. The experiments involving zebrafish were performed under the approval of the Institutional Animal Care and Use Committee of the Institute of Hydrobiology, Chinese Academy of Sciences.

### Transgenic Construct

We download the genomic sequence of *piwil1* from Ensembl (ENSDARG00000041699) and designed the primer pairs to amplify the 5 kb transcriptional regulatory element containing the exon 2 and its upstream sequence according to a previous report (Leu and Draper 2010). The forward primer is 5′-CAAAGAACGAACACCTGGTTGG-3′ and the reverse primer is 5′-ACCGGTGCTTTACAAATGCTGA-3′. The 5-kb fragment was inserted into the upstream of EGFP coding sequence of construct pTol2(EGFP-UTRnanos3) (Kwan et al. 2007; Xiong et al. 2013) to generate the transgenic plasmid, pTol2(piwil1:EGFP-UTRnanos3). The transgenic construct of pTol2(UAS:buc-UTRnanos3) was made based on the pTol2(UAS:mRFP-UTRnanos3) (Xiong et al. 2013). The CDS region of *bucky ball* (*buc*) was amplified from zebrafish ovary cDNA and inserted into the pTol2(UAS:mRFP-UTRnanos3) construct to replace the *mRFP* coding sequence. In-fusion cloning kit (TaKaRa) was used for plasmid construction. All constructs were verified by Sanger sequencing.

### Generation of Transgenic Fish

To generate transgenic zebrafish *Tg*(*piwil1*:*egfp-UTRnanos3*), 50 ng of transgenic construct pTol2(piwil1:egfp-UTRnanos3) was injected into the animal pole of 1-cell embryo and 100 pg transposase mRNA was injected into the yolk. The F0 embryos were screened under fluorescence stereomicroscope (AF205, Leica). The embryos with bright fluorescence were picked and raised up. The F0 fish were mated with wildtype fish to generate F1 fish. The 2 dpf F1 embryos with bright EGFP-labeled PGCs were selected to raise up. At 14 dpf, the F1 larvae were screened to exclude non-specific EGFP expression at other parts of the body. The F1 adult fish were mated with wildtype fish to generate F2 transgenics. The F2 embryos were also screened at 2 dpf and 14 dpf to exclude non-specific EGFP labeling. Finally, the *Tg*(*piwil1*:*egfp-UTRnanos3*)^*ihb327Tg*^ was established and deposited in CZRC.

The transgenic line *Tg*(*UAS*:*buc-UTRnanos3*)^*ihb120Tg*^ was generated with a similar method by injection of the pTol2(UAS:buc-UTRnanos3) construct; both F0 and F1 descendants were screened by PCR for the junction of *buc* and *nanos3* 3′-UTR with primers listed in Table 1. The transgenic fish *Tg*(*kop*:*KalTA4-UTRnanos3*,*CMV*:*EGFP*)^*ihb8Tg*^ was described in our previous study and obtained from CZRC (catalog ID: CZ20) (Xiong et al. 2013). To obtain PGC-specific *buc*-overexpressed embryos, we crossed *Tg*(*UAS*:*buc-UTRnanos3*)^*ihb120Tg*^ males with *Tg*(*kop*:*KalTA4-UTRnanos3*,*CMV*:*EGFP*)^*ihb8Tg*^ females.

**Table 1:** RT-qPCR primer list

*nanos3* Fwd: AGACGTGATGTGCCCGTATC Rev: TGATTTGGCGTACACCGAGC	
*piwil1* Fwd: TGACATAACAGATGGCAACCA Rev: GCCCTCTCTCTGTTCAGGACT	
*piwil2* Fwd: TGACAAACAGAGACTGGGTG Rev: TCATACCTCTGCAGGTGGTC	
*vasa* Fwd: GCTCCCACCAGAGAACTTATCAATC Rev: GCAATCTTCCAGGAGTAGCACACAG	
*dnd* Fwd: TGATTCCTCAACCCACCATAA Rev: TGGACTTCATATTGCGGAGA	
*rbpms2b* Fwd: ATAGCACGGGACCCATTCAC Rev: CATCACCTCTGACTCCCAGC	
*buc-CDS* Fwd: CAAGTTACTGGACCTCAGGATC Rev: GGCAGTAGGTAAATTCGGTCTC	
*buc-UTRnanos3* Fwd: TTGATGCTCCGGGAGATTTG Rev: CTGCTGGCTTGTGTACAAG	

### Reverse-Transcription Quantitative PCR Analysis

Embryos at 1 dpf were collected for RNA extraction and reverse transcription. The BioRad CFX Connect Real-Time System was used for transcript quantification. Samples were tested in triplicate for each gene, and resultant *C*_q_ values were averaged. Primer efficiencies and gene expression levels were calculated according to the previous study (Pfaffl 2001). *β-actin* was selected as a reference gene. Data was processed using 2^−ΔΔCq^ method. All reverse-transcription quantitative PCR (RT-qPCR) gene-specific primers are listed in Table 1.

### Whole-Mount In Situ Hybridization

Digoxigenin-labeled antisense RNA probe of zebrafish *vasa* was synthesized by in vitro transcription. Whole-mount in situ hybridization (WISH) was performed as described (Wei et al. 2014).

### Whole-Mount Immunofluorescence

Whole-mount immunofluorescence was performed as described previously with some modifications (Ye and Lin 2013). Incubation of the first antibody was performed at 4 °C overnight. Incubation of secondary antibody was performed at room temperature (23 °C) for 3 h. Samples were immerged in 50% glycerol-PBS at 4 °C overnight before confocal microscopy. The rabbit polyclonal antibody against zebrafish Vasa was used (GTX128306, Gentex, 1:200). Anti-rabbit Alexa Fluor 568 was used as secondary antibody (Molecular Probes).

### Confocal Microscopy

Transgenic larvae at specific stages were dissected by fine tweezers (5SA-JP, VETUS). The gut and liver were removed to expose the swim bladder. The gonad was just located on both sides of the swim bladder. The swim bladder exposed larvae were fixed in 4% methanol-free PFA at 4 °C overnight. After washing with 1× PBS/Triton X-100 (0.1%), the gonads were isolated under fluorescence stereomicroscope (AF205, Leica). The gonads were stained by phalloidin-Alexa Fluor 568 (Molecular Probes) and DAPI, to visualize F-actin and nuclear DNA, respectively. The gonads were then infiltrated by 50% glycerol-PBS at 4 °C overnight. Confocal images were acquired using a laser-scanning confocal inverted microscope (SP8, Leica) with a LD C-Apo 40×/NA 1.1 water objective. Z-stacks were generated from images taken at 0.5-μm intervals, using the following settings (2048 × 2048 pixel, 400 MHz). EGFP intensity and nuclear diameter were measured by Fiji software (Schindelin et al. 2012). The Cell Counter plugin was used to count the number of oogonia, oocyte IA, and oocyte IB. All data were exported to Excel for statistical analysis. The graphs were generated and the Student *t* test was performed using GraphPad Prism software.

## Results

### Lifetime Labeling of Zebrafish Germ Cells in *Tg*(*piwil1*:*egfp-UTRnanos3*)^*ihb327Tg*^

**Fig. 1:**
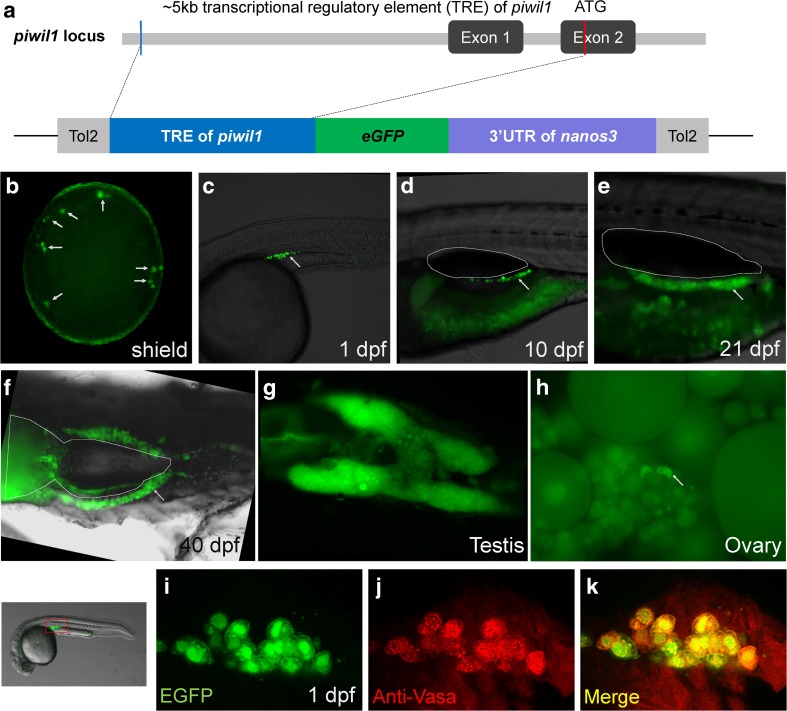
Generation and characterization of *Tg*(*piwil1*:*egfp-UTRnos3*)^*ihb327Tg*^. **a** The structure of the transcriptional regulatory region of *piwil1* locus of zebrafish and the sketch map of transgenic elements. **b–h** Germ cell-specific expression at various developmental stages as indicated in the figure (arrows indicate the EGFP-positive germ cells; outlines indicate the swim bladder). **g** Dissected adult testis. **h** Dissected adult ovary (arrows indicate EGFP-positive oogonia or early stage of oocytes). **i–k** Immunofluorescence of Vasa in the transgenic embryo at 1 dpf. The selected region for imaging is indicated by a red box. **i** EGFP; **j** anti-Vasa; **k** merged channel

To specifically label the germ cells from the embryonic stage to adulthood of zebrafish, we generated a transgenic line using *piwil1* promoter and *nanos3* 3′-UTR to direct the expression of EGFP (Fig. 1a). The PGC-specific EGFP could be detected at shield stage in the embryos derived from transgenic females (Fig. 1b). The early specific labeling of PGCs could be due to the combined effects of maternal transcriptional activity of *piwil1* and post-transcriptional regulation of *nanos3* 3′-UTR. However, the transgenic embryos derived from paternal transgenics only showed PGC-specific EGFP expression after 3 dpf (data not shown), which was likely ascribed to the zygotic transcription driven by *piwil1* promoter. To our surprise, the transgenic line specifically labeled the germ cell lineage throughout the whole lifetime, i.e., from the PGCs at shield stage to the differentiated gametes at adulthood (Fig. 1b–h). Before 21 dpf, the in vivo development of zebrafish germ cells could be observed under fluorescence microscope (Fig. 1b–e). However, as the fish developed until juvenile stage around 25 dpf, the pigments and adipocyte enriched at the region of gonad; thus, it was difficult to focus the gonad under a microscope. However, the fluorescent gonad could be still seen when the fish was dissected (Fig. 1f). In adult fish, the whole testis showed bright fluorescence while the ovary appeared to show EGFP expression in oogonia and early stage of oocytes (Fig. 1g, h).

To verify that the EGFP specifically labeled the PGCs at the embryonic stage, we performed immunofluorescence using Vasa antibody on transgenic embryos at 1 dpf. As shown in Fig. 1 i–k, the Vasa signals were perfectly co-localized with EGFP, suggesting that the EGFP were exclusively expressed in the PGCs. Taken together, we successfully generate a transgenic line which labels germ cell lineage throughout the whole lifetime of zebrafish.

### Early Dimorphic Gonads Lead to Male- or Female-Biased Development

**Fig. 2:**
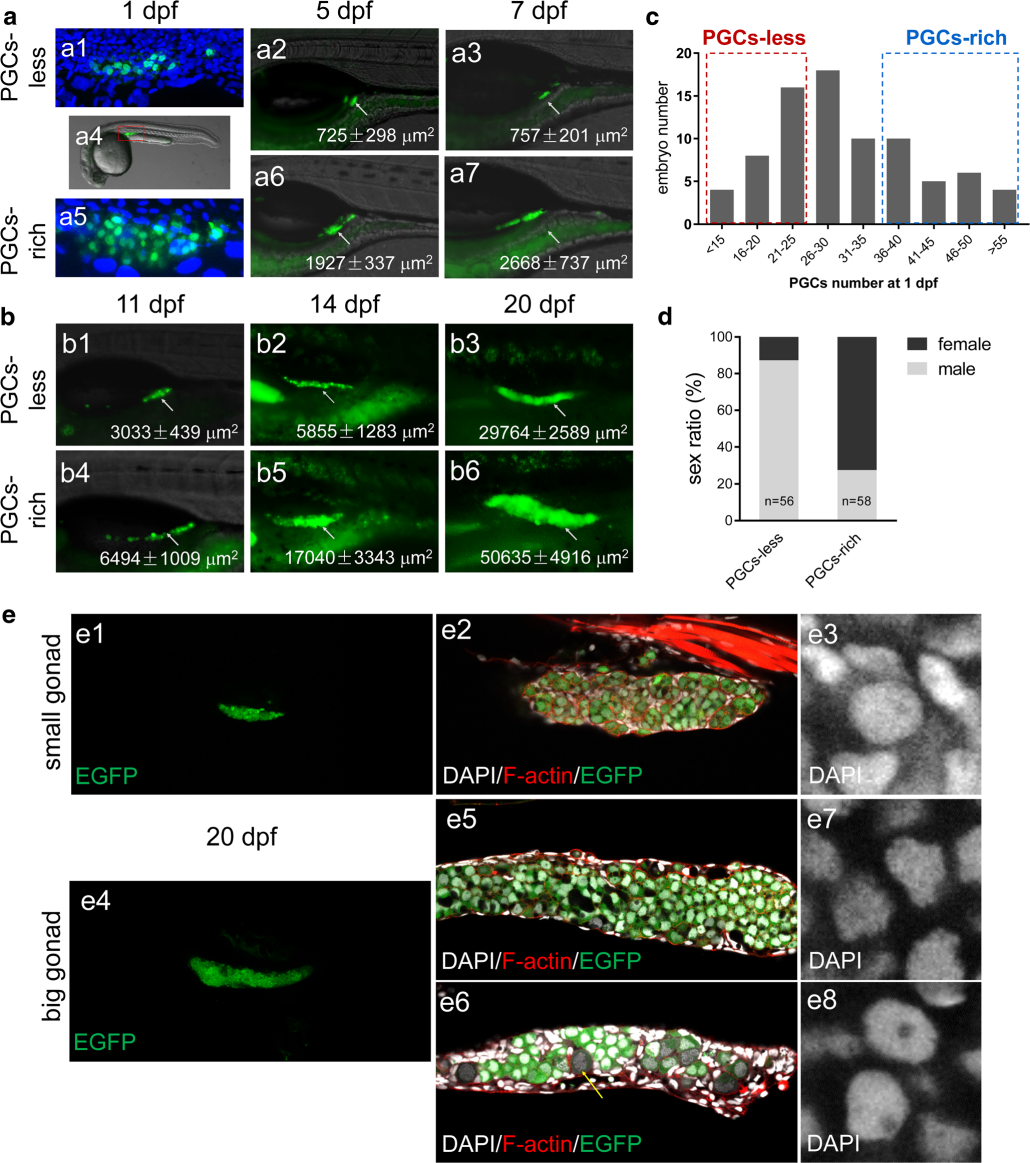
Using live tracing to study the correlation between the initial PGC numbers and sexual development. **a** Representative images of PGC-less and PGC-rich embryos at 1 dpf (a1, a5), 5 dpf (a2, a6), and 7 dpf (a3, a7). **b** Representative images of PGC-less and PGC-rich larva fish at 11 dpf (b1, b4), 14 dpf (b2, b5), and 20 dpf (b3, b6). **c** Frequency distribution of PGC number at 1 dpf; two boxes with dashed frame label the selected population of “PGC-less” and “PGC-rich.” The lateral areas of gonads were calculated and were shown at the lower right corner of images in **a** and **b**. **d** The sex ratio in the population of PGC-less and PGC-rich embryos. **e** Confocal microscopy of small and big gonads at 20 dpf with *Tg*(*piwil1*:*egfp-UTRnos3*)^*ihb327Tg*^ transgenic fish. **e1** Confocal image of small gonads. **e2** Higher magnification of a representative image of small gonad displaying combined channels of DAPI, F-actin, and EGFP. **e3** Magnification image showing the nuclei of gonocyte in a small gonad. **e4** Confocal image of big gonads. **e5**, **e6** Higher magnification of representative images of big gonads displaying combined channels of DAPI, F-actin, and EGFP. **e5** shows an example in which the germ cells have an irregular shape of nuclei; **e6** shows an example in which chromatin nucleolus-stage oocytes exist. **e7** Magnification image showing cells with irregular shape of nuclei. **e8** Magnification image showing that the nuclei of gonocyte were similar to the one in a small gonad

In previous studies, it has been shown that the number of undifferentiated germ cells plays an important role in zebrafish sexual differentiation (Tong et al. 2010; Tzung et al. 2015). To further investigate the correlation of early PGC number and sexual differentiation, we carefully trace the number of PGCs or undifferentiated germ cells from the embryonic stage to larval stage. To accurately count the PGC number at 1 dpf, we utilized optical sectioning of confocal microscopy (Fig. 2a). Interestingly, the embryonic PGC numbers at 1 dpf were variable from 12 to 70 (Fig. 2a, c). We carefully selected the embryos with a low amount of PGCs (less than 25) and the embryos with a high amount of PGCs (more than 36) and raised them in two groups. After the embryos developed after 5 dpf, we took photos for gonads of each group of fish. As expected, the variation on PGC number between the two groups persisted (Fig. 2a) and led to primitive gonads with dimorphic sizes (small gonad and big gonad) at different larval stages (Fig. 2b). These two groups were then raised up and their sex ratios were measured at adulthood. We found that the group with a low amount of PGCs grew up into the male-bias population (80.8% males) while the group with a high amount of PGCs grew up into the female-bias population (72.4% females) (Fig. 2d, data collected from three independent experiments). Taken together, these data reveal that previously unappreciated variation on PGC number occurs at the early embryonic stage, and high amount of PGCs facilitate female differentiation.

The dimorphic sizes became more obvious at 20 dpf. To carefully examine the cellular characters of both small and big gonads, we performed confocal microscopy (Fig. 2e (e1, e4)). Most of the germ cells in both small and big gonads were gonocytes with a visible nucleolus (Fig. 2e (e3, e8)). Occasionally, stage IA oocytes could be seen in big gonads (Fig. 2(e6) “arrow”). Interestingly, we observed that the nuclei of most germ cells in big gonad were of irregular shape rather than round shape while the nuclei in most of the germ cells in small gonad were round (Fig. 2(e2, e3 e5, e7)). These data suggest that small and big gonads may undergo different morphogeneses of germ cells, leading to a phenotype of gonadal dimorphism from 20 dpf.

### Abundance of Early PGCs Promotes Female Development

**Fig. 3:**
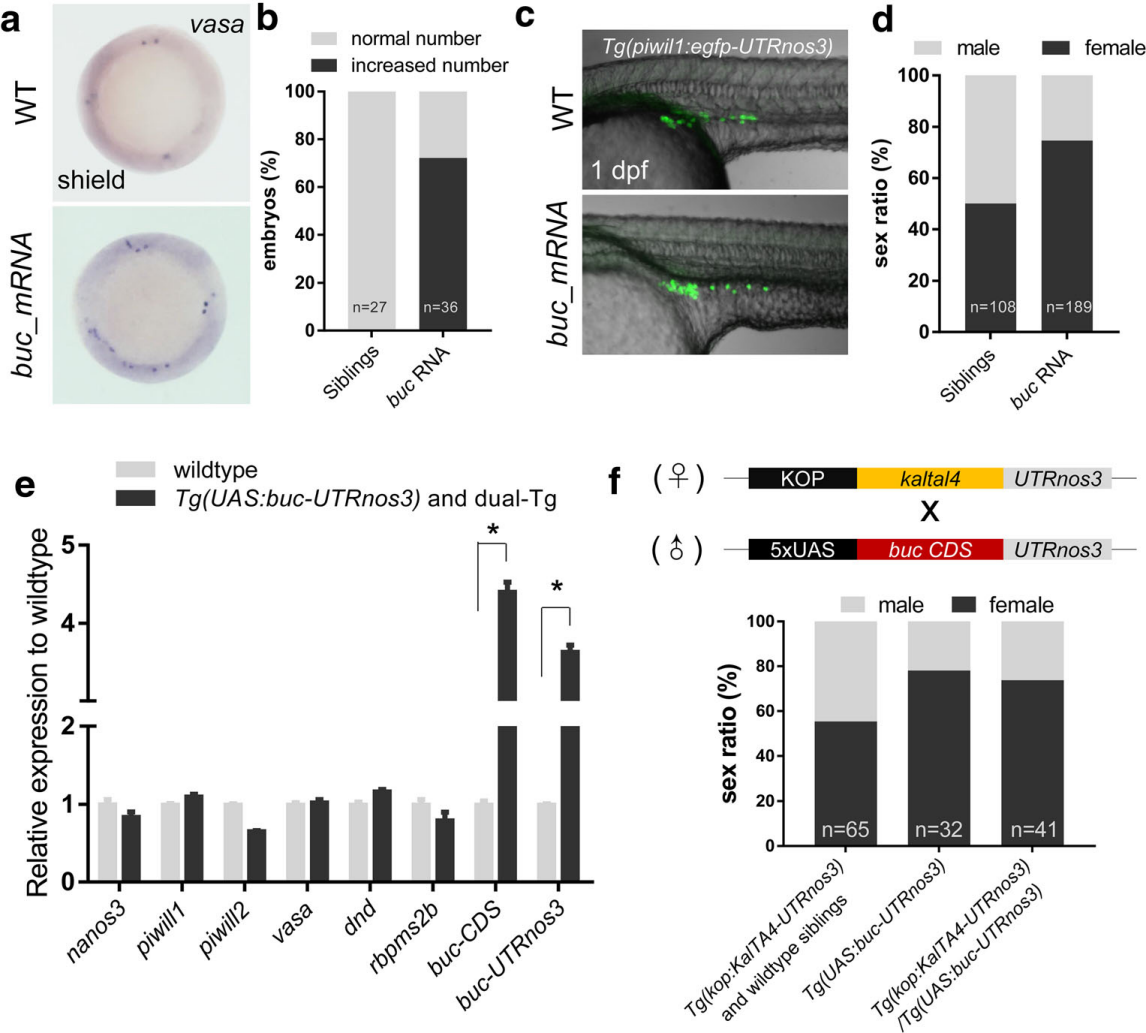
The abundance of early PGCs promotes female development. **a** Representative images showing PGCs in wildtype and *buc*-overexpressed embryos at shield stage (PGCS was visualized by WISH). **b** Percentage of embryos with increased PGCs or normal number of PGCs in wildtype and *buc*-overexpressed embryos. **c** Representative images showing PGCs in wildtype and *buc*-overexpressed embryos at 1 dpf (PGCS was visualized using *Tg*(*piwil1*:*egfp-UTRnanos3*)^*ihb327Tg*^). **d** The sex ratio in population of wildtype and *buc*-overexpressed embryos. **e** RT-qPCR showing that *buc* was overexpressed in *Tg*(*kop*:*KalTA4-UTRnanos3*,*CMV*:*EGFP*)^*ihb8Tg*^*/Tg*(*UAS*:*buc-UTRnanos3*)^*ihb120Tg*^ double-transgenic fish (dual-Tg) and *Tg*(*UAS*:*buc-UTRnanos3*)^*ihb120Tg*^. **f** The sex ratio in the populations of sibling control (*Tg*(*kop*:*KalTA4-UTRnanos3*,*CMV:EGFP*)^*ihb8Tg*^ and wildtype fish), *Tg*(*UAS*:*buc-UTRnanos3*)^*ihb120Tg*^ and double transgenics

In the previous study, it has been shown that overexpression of *buc* generates ectopic germ cells in zebrafish embryos (Bontems et al. 2009). To investigate whether increasing embryonic PGC number would promote the female differentiation, we injected *buc* mRNA into a zygote and successfully induced more PGCs at shield stage (Fig. 3a, b). The injected embryos were raised up to adulthood and their gender was identified. Interestingly, we found that the ratio of females was significantly higher than that of the uninjected siblings (Fig. 3c), with 74.7% female in *buc*-overexpressed population vs 50.1% of female in the uninjected siblings. These data strongly suggest that induction of embryonic PGCs promotes female differentiation.

To further verify the above finding, we performed PGC-specific overexpression of *buc* using Gal4/UAS inductive overexpression method by crossing heterozygote *Tg*(*kop*:*KalTA4-UTRnanos3*,*CMV*:*EGFP*)^*ihb8Tg*^ females with heterozygote *Tg*(*UAS*:*buc-UTRnanos3*)^*ihb120Tg*^ males. All the offspring with mixed genetic background were raised together in the same tank until adulthood. Their genotypes and genders were individually identified either by PCR or by expression of EGFP. To verify that *buc* was overexpressed in the double transgenics, we performed RT-qPCR analysis on 1 dpf embryos of double-transgenic fish and *Tg*(*UAS*:*buc-UTRnanos3*)^*ihb120Tg*^ using the primer pairs amplify the region covering the junction of *buc* and *nanos3* 3′-UTR (*buc-UTRnanos3*) (Fig. 3d). The expression of both the coding region of *buc* and the junction of *buc-UTRnanos3* was remarkably increased in the *Tg*(*UAS*:*buc-UTRnanos3*)^*ihb120Tg*^ or double-transgenic fish when compared with the wildtype siblings (Fig. 3d). Similar to the result from *buc* injection, the double-transgenic siblings showed female bias compared to wildtype siblings (Fig. 3e), and the *Tg*(*UAS*:*buc-UTRnanos3*)^*ihb120Tg*^ siblings also showed pro-female development, due to the reason that the maternally deposited Gal4 proteins in the eggs of heterozygote *Tg*(*kop*:*KalTA4-UTRnanos3*) female could activate the *buc* expression in *Tg*(*UAS*:*buc-UTRnanos3*)^*ihb120Tg*^ embryos. Taken together, our data demonstrate that abundant embryonic PGCs facilitate the female differentiation.

### Ovary Development in *Tg*(*piwil1*:*egfp-UTRnanos3*)^*ihb327Tg*^

**Fig. 4:**
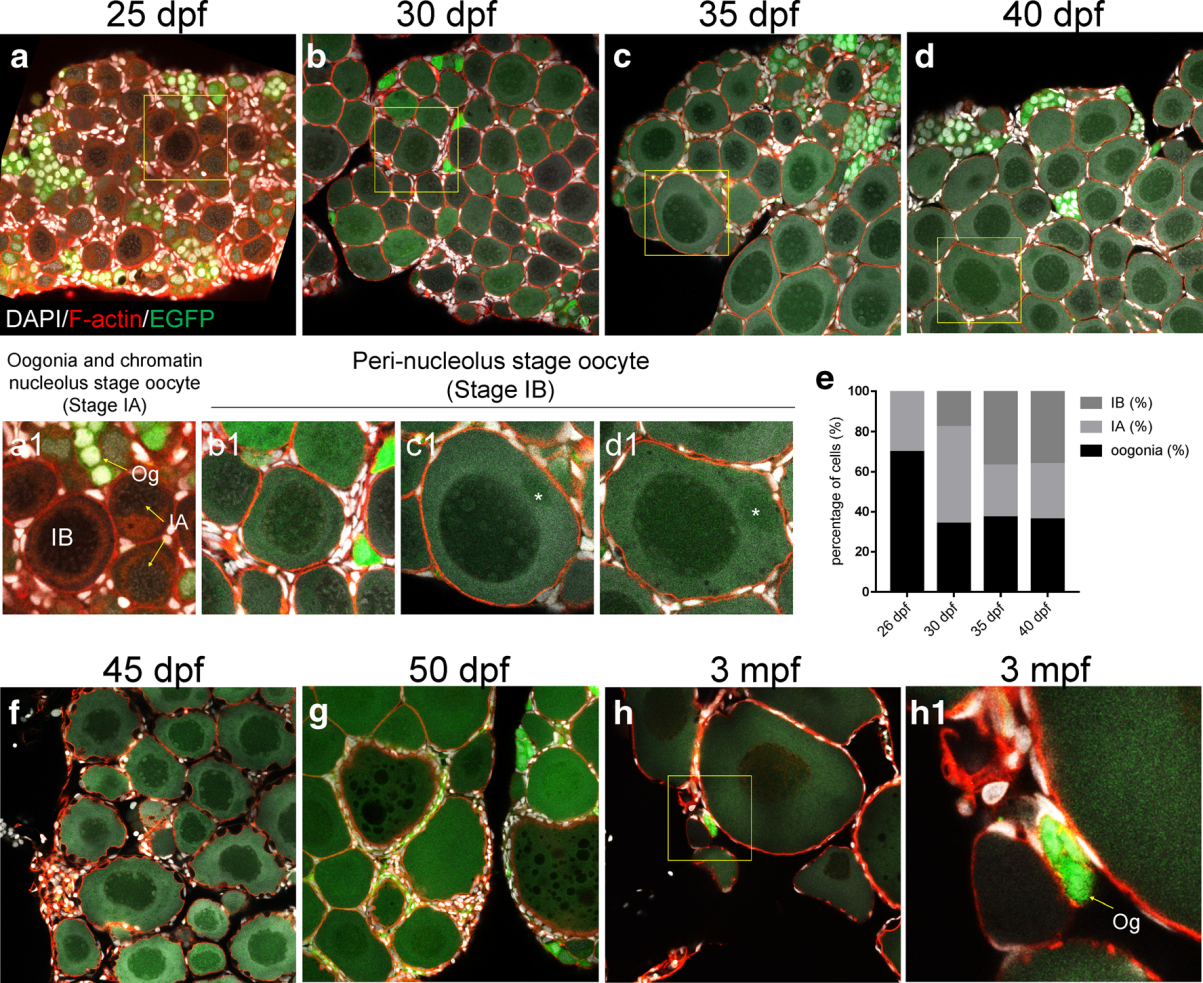
Confocal microscopy of ovary development. **a**–**h** Confocal images of developing ovaries at 25 dpf (**a**), 30 dpf (**b**), 35 dpf (**c**), 40 dpf (**d**), 45 dpf (**e**), 50 dpf, and 3 mpf (**f**). **a1**, **b1**, **c1**, **d1**, and **h1** are the magnified regions of interest in **a**, **b**, **c**, **d**, and **h** respectively. **e** The graph showing the percentage of oogonia, stage IA oocyte, and stage IB oocyte in ovaries of 26 dpf, 30 dpf, 35 dpf, and 40 dpf. Arrows in **a1** indicate the oogonia (Og), stage IA oocyte (IA), and stage IB oocyte (IB); asterisk in **c1** and **d1** indicates the Balbiani body; arrow in **h1** indicates the oogonia

The development of ovary from 25 to 50 dpf was carefully assessed using *Tg*(*piwil1*:*egfp-UTRnanos3*)^*ihb327Tg*^. A few stage IB oocytes started to be visible in big gonad at 25 dpf (Fig. 4a (a1)). There were mainly oogonia, stage IA oocyte, and stage IB oocyte in the ovary from 25 to 40 dpf (Fig. 4a–d (a1–d1)). From middle to late stage of IB oocytes, the Balbiani body could not be stained by DAPI and formed a dark dot (Fig. 4(c1, d1) asterisk). As shown in Fig. 4 e, from 25 to 35 dpf, the percentage of oogonia decreased while the percentage of stage IA and stage IB oocytes increased, suggesting proceeding of oogenesis from oogonia to stage I oocyte. From 35 to 40 dpf, the portion of each cell type was fairly stable (Fig. 4e). At 40 dpf, oocytes transiting from stage IB to stage II began to be visible (Fig. 4d (d1)). Strikingly, at 45 dpf, most of the stage IB oocytes started to develop into stage II oocytes in a simultaneous manner (Fig. 4f). At 50 dpf, the stage II oocytes started to be visible which were marked by thick cortical actin and robust increasing of cortical alveolus (Fig. 4g). It was worth noting that the EGFP was persistently expressed in oogonia from 25 dpf to 3 mpf (Fig. 4a–h). Thus, the whole process of ovary development can be visualized at intact-gonad level with high resolution using *Tg*(*piwil1*:*egfp-UTRnanos3*)^*ihb327Tg*^.

### Testis Development in *Tg*(*piwil1*:*egfp-UTRnanos3*)^*ihb327Tg*^

**Fig. 5:**
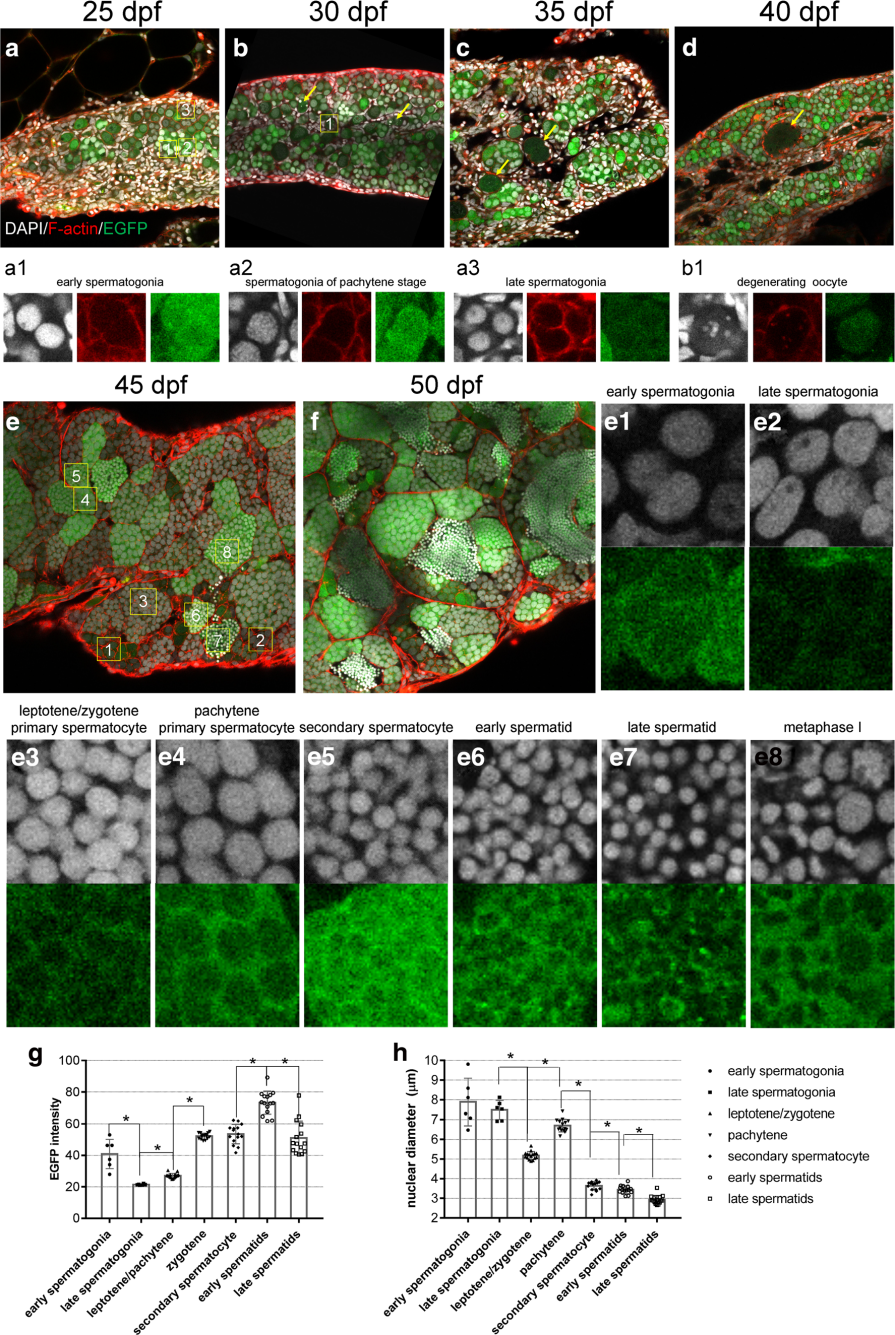
Confocal microscopy of testis development. **a**–**d** Confocal images of developing testes at 25 dpf (**a**), 30 dpf (**b**), 35 dpf (**c**), and 40 dpf (**d**). Arrows in **b**, **c**, and **d** indicate the degenerating oocytes. **a1**–**a3** are the magnified regions of interest labeling **1** to **3**, respectively, in **a**; **b1** is the magnified region of interest labeling **1** in **b**. **a1**–**a3** Representative image showing EGFP-positive early spermatogonia (**a1**), EGFP-positive early spermatogonia of Pachytene stage (**a2**), and weak EGFP in late spermatogonia (**a3**) in **a**. **b1** Representative image showing degenerating oocyte in **b**. **e**–**f** Confocal images of developing testes at 45 dpf (**e**) and 50 dpf (**f**). **e1**–**e8** are the magnified regions of interest labeling **1** to **8**, respectively, in **e**. **e1**–**e8** Representative images showing early spermatogonia (**e1**), late spermatogonia (**e2**), primary spermatocyte at leptotene/zygotene stage (**e3**), primary spermatocyte at pachytene stage (**e4**), secondary spermatocyte (**e5**), early spermatid (**e6**), late spermatid (**e7**), and primary spermatocyte at metaphase I (**e8**). **g** Graph showing EGFP intensity of various types of spermatogenic cells in **e**. **h** Graph showing the nuclear diameter of various types of spermatogenic cells in **e**. The left, middle, and right images in **a1**–**a3** and **b1** showed DAPI, F-actin, and EGFP, respectively; upper row in **e1**–**e8**: DAPI; lower row in **e1**–**e8**: EGFP; Asterisk indicates *P* value < 0.01

The development of testis from 25 dpf to 50 dpf was carefully assessed using *Tg*(*piwil1*:*egfp-UTRnanos3*)^*ihb327Tg*^. Unlike the small gonad at 21 dpf, the germ cells in the small gonad at 25 dpf were mainly different subtypes of spermatogonia with different nuclear sizes and fluorescent intensities, indicating proceeding of spermatogenesis at this stage (Fig. 5a (a1–a3)). The degeneration of oocytes occurred at 30 dpf and ended at 40 dpf (Fig. 5b–d (b1)). Unlike that the oocytes which were surrounded by follicle cells, the germ cells in testis aggregated into cell clusters (Fig. 5a–f). Strikingly, spermatid could be seen at as early as 45 dpf, indicating that the whole process of spermatogenesis has completed at this time (Fig. 5e). At 50 dpf, testis developed morphologically identified tubular compartment which contained germ cells of different stages of spermatogenesis (Fig. 5f).

Visualization of the process of testis development and specific labeling of germ cells by *Tg*(*piwil1*:*egfp-UTRnanos3*)^*ihb327Tg*^ provided us an opportunity to identify different cell types in intact gonads during spermatogenesis. The different germ cell stages during spermatogenesis are generally divided into main four classes—spermatogonia, primary spermatocytes, secondary spermatocytes, and spermatid (Schulz et al. 2010). We carefully measured diameters of cell nuclei and intensities of different germ cells to characterize germ cells of different developmental stages from 45 dpf (Fig. 5 e(e1–e8)).

Based on both nuclear morphology and EGFP intensity of germ cells on a 45 dpf testis, there are two subtypes of spermatogonia—one with less number and higher EGFP intensity, whereas the other with more cells but lower EGFP intensity. However, there is no significant difference in nuclear diameter between early and late stages of spermatogonia (Fig. 5 e(e1, e2)). Our observation indicates that the spermatogonia with lower EGFP intensity is at a later stage and the one with higher EGFP intensity is at an earlier stage which might be the type A spermatogonia. As meiotic prophase proceeded, the intensity of EGFP increased and reached a peak at an early stage of spermatid, and the average intensity decreased but become aggregated in the late stage of spermatid (Fig. 5 e(e3–e8), g, h). Taken together, the early development of testis and spermatogenesis can be characterized in details at intact-gonad level using *Tg*(*piwil1*:*egfp-UTRnanos3*)^*ihb327Tg*^.

**Fig. 6:**
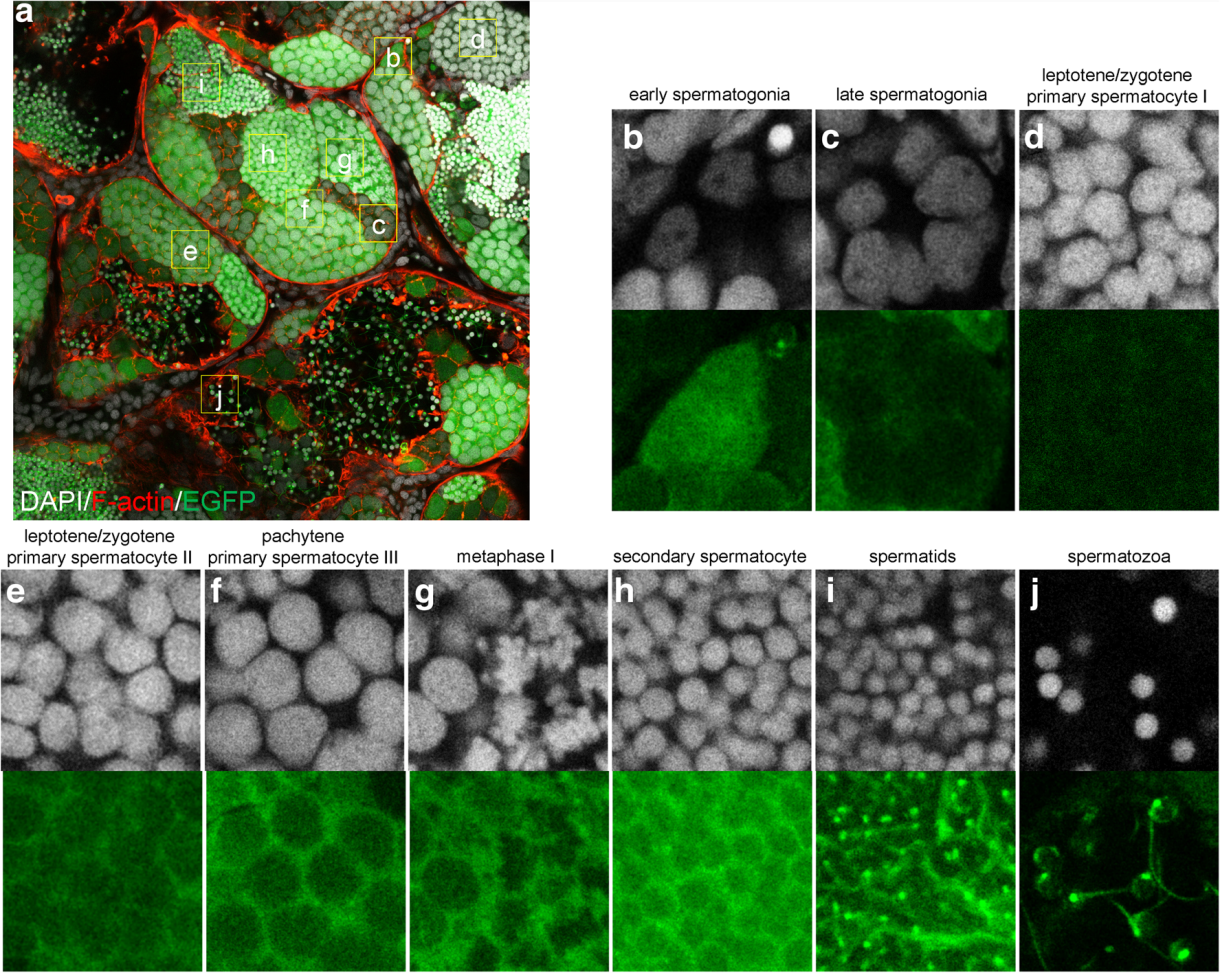
Confocal microscopy of adult testis. **a** Confocal image of adult testis. **b**–**j** The magnified regions of interest in **a**; **b**–**j** Representative images showing early spermatogonia (**b**), late spermatogonia (**c**), primary spermatocyte at leptotene/zygotene stage (**d**), primary spermatocyte at late leptotene/zygotene stage based on the EGFP intensity (**e**), primary spermatocyte at pachytene stage (**f**), primary spermatocyte at metaphase I (**g**), secondary spermatocyte (**h**), spermatid (**i**), and spermatozoa (**j**). Upper row in **b**–**j**: DAPI; lower row in **b**–**j**: EGFP

To verify whether the EGFP intensity can be used as a reliable indicator for identification of germ cells at different stages in the matured testis, we performed an analysis in the testis of adult zebrafish at 5 mpf (Fig. 6a). Consistently, we could identify early and late spermatogonia based on their EGFP intensities (Fig. 6b, c). The expression of EGFP also increased during the meiotic prophase and reached the highest expression at secondary spermatocytes (Fig. 6d–h). More interestingly, we found that the EGFP aggregated to the posterior region of the head of spermatozoa and specifically labeled its tail (Fig. 6i, j). Taken together, using EGFP intensity as a criterion, we could easily identify the germ cells at different stages during spermatogenesis in the *Tg*(*piwil1*:*egfp-UTRnanos3*)^*ihb327Tg*^.

## Discussion

Transgenic labeling of germ cells by fluorescent proteins provides us with a powerful tool to study the germ cell development and gonad differentiation of teleost. In the present study, we have successfully generated a transgenic zebrafish line *Tg*(*piwil1*:*egfp-UTRnanos3*)^*ihb327Tg*^, in which the lifetime germline could be specifically and efficiently labeled for the first time. By using this transgenic line, we were able to visualize the lifetime development of zebrafish germline and to analyze the relationship between initial embryonic PGC number and the later gonad differentiation.

In the previous studies, although different types of germ cells at different stages have been labeled by various transgenic zebrafish, none of them could label the whole lifetime of zebrafish germline. For instance, a similar transgenic line, *Tg*(*piwil1*:*EGFP*)^*uc1Tg*^ could label the germline from 7 dpf but not the embryonic PGCs before that, probably due to that a different SV40 polyA signal was used in their study (Leu and Draper 2010). In our transgenic fish, the use of *piwil1* promoter ensures the maternal expression of EGFP and the use of 3′-UTR of *nanos3* ensure its restricted expression in PGCs. Thus, the embryonic PGCs could be labeled as early as the shield stage. During spermatogenesis, unlike the *Tg*(*piwil1*:*EGFP*)^*uc1Tg*^, which showed highest EGFP expression level in spermatogonia (Leu and Draper 2010), our *Tg*(*piwil1*:*egfp-UTRnanos3*)^*ihb327Tg*^ displayed different expression levels in different types of spermatogenic cells, with a relatively high level in spermatocyte and spermatid. Considering that the *Tg*(*piwil1:egfp-UTRnanos3*)^*ihb327Tg*^ has specific expression pattern on labeling different types of germ cells particularly the germline stem cells, it will be of great interest to use this transgenic fish to identify and sort out the early type A spermatogonia, oogonia, and PGCs combining the usage of fluorescent-activated cell sorting (FACS) in the future. Those sorted germline stem cells can be used not only for transcriptome analysis but also for research on surrogate reproduction (Morita et al. 2015).

It is reported that the number of PGCs plays critical roles in sexual development and the depletion of PGCs usually results in infertile male in some fish species such as zebrafish, medaka, and *Carassius gibelio* (Kurokawa et al. 2007; Liu et al. 2015; Siegfried and Nusslein-Volhard 2008). However, there is no report of the effects of early germ cell induction on later sex differentiation. In our present study, we utilized the *Tg*(*piwil1*:*egfp-UTRnanos3*)^*ihb327Tg*^ transgenic line to unveil that rich embryonic PGC number led to later ovary differentiation and female development and less embryonic PGC number resulted in later male development. More interestingly, we utilized the method of *buc* overexpression (Distel et al. 2009), by either transient overexpression with mRNA injection or stable overexpression with Gal4/UAS transgenic system (Xiong et al. 2013) to induce ectopic PGCs at shield stage. The induction of PGCs at shield stage could also promote female differentiation. All these data demonstrate that the initial number of PGCs should greatly contribute to the sex differentiation in zebrafish. In the future, it will be interesting to test whether different amounts of germplasm exist in different oocytes and which mechanism is responsible for this type of germplasm variation. Since the Gal4/UAS system has been successfully utilized for PGC-specific overexpression of *buc* which leads to female-biased development, it might have great application in female-biased breeding by induction of female development in some commercial fish species with sexual growth dimorphism (Mei and Gui 2015).
